# Perception of National Youth Service Corps (NYSC) among corps medical doctors in Nigeria: a cross-sectional study

**DOI:** 10.1186/s12909-023-04135-1

**Published:** 2023-03-15

**Authors:** Chibuike Emmanuel Nwachukwu, Edward Oluwatobi Olufunmilayo, Gideon Bulus Chiroma, Chukwuebuka Francis Okoye

**Affiliations:** 1 St Leo’s Hospital, Enugu, Enugu State Nigeria; 2 Abia State Specialist hospital, Amachara, Umuahia, Abia State Nigeria; 3 Health Services Department, Ignatius Ajuru University of Education, Port-Harcourt, Rivers State Nigeria; 4 Nigerian Air Force Military Hospital, Jos, Plateau State Nigeria

**Keywords:** National Youth Service Corps, Doctors, Benefits, Challenges, Treatment, Nigeria

## Abstract

**Background:**

The National Youth Service Corps (NYSC) is a compulsory one-year programme for graduates of tertiary schools including doctors. This study was conducted to find out the benefits and challenges of the programme among corps medical doctors as well as their treatment in their places of primary assignment (PPAs). The study would influence policymaking so as to improve the experiences of corps medical doctors and help NYSC to achieve its objectives.

**Methods:**

A descriptive cross-sectional survey was conducted among 399 medical doctors doing their compulsory national youth service in Nigeria. The research instrument was developed by the researchers and pretested before use. The outcome variables were: overall benefit, overall challenge and overall PPA treatment. The data obtained were analyzed using descriptive statistics and chi-square test using p < 0.05.

**Results:**

Majority of respondents (83.5%) believed that the scheme offered one or more benefits to corps medical doctors (overall benefit). These benefits include exposure to new culture (47.6%), gaining medical/surgical skills (36.3%) and finding a lover/spouse (4.5%). Similarly, most of respondents (89.2%) reported one or more challenges (overall challenge). Some of the challenges reported include; no provision of accommodation (51.6%), poor remuneration (44.7%) and exploitation of corps doctors by their employers (41.4%). There was a statistically significant association between overall benefit and region of deployment (p value: 0.013). Region of deployment and marital status were found to have a statistically significant association with the overall challenge with p-values of 0.031 and < 0.001 respectively. Overall treatment at PPAs was mostly reported to be bad (77.2%) and it had a statistically significant association with marital status (p-value: 0.002) and religion (p-value: 0.024).

**Conclusion:**

Most respondents reported poor PPA treatment and this calls on the government and other stakeholders to take drastic measures to improve the welfare of corps medical doctors in order to positively influence their perception of the scheme and encourage their continued participation.

**Supplementary Information:**

The online version contains supplementary material available at 10.1186/s12909-023-04135-1.

## Introduction

The National Youth Service Corps (NYSC) was established by decree No.24 of 22nd May 1973 [1] and since its inception, the programme has been met with criticism from participants (corps members), who have identified problems faced by corps members including medical doctors [2–4]. These problems are likely to influence the performance of corps medical doctors during the scheme. The NYSC was established in the aftermath of the Nigerian civil war to promote national unity [1]. The programme is run by the NYSC National Directorate Headquarters, a government agency and corps members refer to graduates of universities, polytechnics or other recognized higher institutions including medical doctors [5]. Following graduation from medical school, medical doctors go for a compulsory 1-year internship after which they are eligible to enroll in NYSC programme. The scheme is divided into four parts: orientation, primary assignment, community development service and passing out parade [1]. During mobilisation for the scheme, prospective corps members are expected to choose four states of their choice for possible deployment. Following mobilization, corps members, including doctors, go for 3 weeks of orientation at camps all over the states of the federation, after which they are sent to various facilities for primary assignment and for doctors, a hospital setting.

Some of the problems associated with the programme include being posted to harsh, unfavourable or undesirable locations [4], insecurity due to religious, ethnic and political violence [6, 7], accidents and deaths due to poor transportation networks [8], exploitation and poor welfare at places of primary assignment [9], refusal of relocation requests [10], unavailability of decent accommodation facilities [3, 4] and poor remuneration [11]. Most corps medical doctors earn less than half of what they earned during their internship. This gross reduction in income in spite of the higher skill level afforded by prior internship training could be discouraging and this could lead to poor patient care due to lack of motivation. According to a study done in 2015, the prevalence of anxiety, depression and stress was found to be high among corps members [12]. With studies having shown that mental health problems are more common among medical personnel [13], corps medical doctors are at risk of various psychosocial problems which may or may not be related to their profession. The lack of adequate support structure has been implicated in the poor performance of the scheme [14]. A study by O S Fadairo in 2010 concluded that the influence of the NYSC scheme on the development of youth corps members was perceived to be poor by the majority of the respondents [15]. This study was done among non-medical personnel. Our study is a unique pioneer study, as it seeks to explore the challenges faced by corps medical doctors specifically.

There have been some concerns over the relevance of the scheme. There was a recent bill sponsored by a lawmaker in the national assembly demanding that the scheme be scrapped but the bill is yet to be approved [2]. Despite the challenges bedevilling the scheme, it has not only provided an avenue for corps doctors to gain medical and surgical skills but also an opportunity to help cushion the devastating effects of industrial action by resident doctors in Nigeria [16]. This is beneficial to the doctors as well as the general public as these doctors develop more confidence in patient care.

A study focused on the experiences of corps medical doctors in Nigeria does not currently exist. The challenges of the scheme faced by the average corps medical doctor go a long way to influence his/her general attitude towards the scheme, mental health status and essentially the quality of medical care they offer to patients in their places of primary assignment. The aim of this study was to determine the benefits and challenges of the NYSC programme among corps medical doctors in Nigeria as well as their treatment at their PPAs. An understanding of the experiences of the service year among corps medical doctors would guide stakeholders on how best to meet the psychosocial needs of corps doctors and this will help in reducing the prevalence of mental health problems among them and potentially improve the quality of care they offer to patients in the communities they get posted to work in.

Similar national youth programmes exist in other countries of the world such as Israel, France, United States of America, Ghana, Uganda, Zimbabwe, South Africa, etc. [17–19], however, the mode of operation, requirements and demographic categories involved in such schemes vary widely among these countries. For example, in Kenya and Zimbabwe, National Youth Service is a voluntary scheme for secondary school leavers [20, 21], implying that the participation of medical doctors in the scheme will be uncommon. These peculiarities have compounded the dearth of data and published works on the perception of National Youth Service programmes among participating doctors in Africa and the world. Therefore, this study will add to the existing literature and fill up the gap created by the paucity of data on the benefits, challenges and PPA experience of corps medical doctors in Nigeria.

## Methodology

This study was carried out among serving corps medical doctors in Nigeria. The NYSC was founded in 1973 for tertiary education graduates including doctors to undergo one-year service to fatherland. About 150,000 corps members are recruited yearly but the exact number of doctors recruited yearly could not be ascertained [22]. Corps members are recruited in 2 to 3 batches per year and each batch has 2 streams. The programme runs in cycles such that as one stream is passing out, another stream is being recruited.

### Study design

A descriptive cross-sectional study was conducted among 399 serving corps doctors at various places of primary assignment across all states in the 6 geopolitical zones of the country, including Abuja.

### Study population

The study was conducted among 399 serving corps doctors. All corps doctors, irrespective of age or sex, serving at the time of the study and gave consent, were recruited for the study. Corps medical doctors who had not completed camp orientation were excluded.

### Sample size

The minimum sample size of 384 was calculated using the Cochran’s formula using the proportion of 50% as the researchers could not find a similar and acceptable study on the subject to adopt a specific prevalence value from.

### Data collection

Data on participants’ socio-demographics as well as the benefits, challenges and treatment at PPA was obtained with a pre-tested, semi-structured, self-administered e-questionnaire (google form) which was developed by the researchers from anecdotal evidence and previous study reports [4]. The authors were assigned geopolitical zones to cover and participants were reached through corps medical doctors’ online platforms, mainly WhatsApp. Initial questions on the google forms were used to screen out those who did not meet the inclusion criteria. All serving corps doctors at various places of primary assignment across all the states in the 6 geopolitical zones of Nigeria, including Abuja, were sampled.

### Data analysis

Data obtained were sorted using Microsoft Excel 2016 and were entered and analyzed using the Statistical Package for Social Sciences (SPSS) version 22. Descriptive statistics such as frequency, percentages and mean with or without Standard deviation were used to summarize and present the results. Chi-square test was used to determine associations between variables.

### Outcome variables

The outcome variables include: overall benefit which comprises the proportion of respondents who reported at least one benefit (Yes) and proportion of respondents who reported no benefit (No); overall challenge which represents the proportion of respondents who reported at least one challenge (Yes) and the proportion of respondents who reported no challenge (No); and overall treatment at PPA which shows the proportion of respondents who reported good treatment at their PPA (Good) and the proportion of respondents who reported bad treatment at their PPA (Bad).

## Results

A total of 399 corps doctors (male: 50.4%, female: 49.6%) were surveyed. The mean age of respondents was: 27.2 years (standard deviation: 1.8) and the majority of them were single (83.3%) and Christians (66.9%). The distribution of the respondents among the major tribes was Hausa (17.5%), Igbo (26.3%) and Yoruba (23.1%).

More than half of the respondents (59.1%) were deployed to serve in the North. About 54% of respondents were not deployed by the NYSC to any of their four most preferred states. Majority of the respondents had spent at least 6 months (63.2%) and were serving in government institutions (82.5%). Table 1.

**Table 1:** Socio-demographic characteristics of respondents

Variable	Response	Frequency (N = 399)	Percentage %
Age (Years) Mean Age: 27.2	23–29	354	88.7
	30 and above	45	11.3
Sex	Male	201	50.4
	Female	198	49.6
Tribe	Hausa	70	17.5
	Igbo	105	26.3
	Yoruba	92	23.1
	Others*	132	33.1
Marital status	Married	67	16.7
	Single	332	83.3
Religion	Christianity	267	66.9
	Islam	132	33.1
Location of medical school	Abroad	70	17.5
	Nigeria	329	82.5
Region of Deployment	North	236	59.1
	South	163	40.9
Deployment to any of the four preferred states	Yes	184	46.1
	No	215	53.9
Length of time in service	Less than 6 months	147	36.8
	6 months and above	252	63.2
PPA Type	Government	329	82.5
	Non-Government	70	17.5

^*Others under tribe represents the minor ethnic groups in Nigeria^

Only 83% of corps doctors were paid by their PPAs and the majority of them (66.9%) were paid less than 80 thousand naira (185 USD) monthly. About half of our respondents (51.6%) were not provided with accommodation by their PPAs. More than half of the participants (65.9%) took call duties at their PPAs with half of them taking at least 3 call duties per week. Majority of the corps medical doctors (79.4%) were unhappy with their work in their PPAs. Most respondents (77.2%) reported overall bad treatment of corps doctors in their PPAs. Table 2.

**Table 2:** Treatment at place of primary assignment

Variable	Response	Frequency (N = 399)	Percentage (%)
Paid by PPA	Yes	331	83.0
	No	68	17.0
Range of pay (Naira)	Less than 80,000	267	66.9
	80,000 and above	64	16.1
	**Not paid**	**68**	**17.0**
Provision of accommodation by PPA	Yes	193	48.4
	No	206	51.6
Take call duty	Yes	263	65.9
	No	136	34.1
If yes, call duty frequency	One-two per week	139	34.8
	Three or more per week	124	31.1
	**No call duty**	**136**	**34.1**
Happy with the job at your PPA	Yes	82	20.6
	No	317	79.4
Overall Treatment at PPA	Good	91	22.8
	Bad	308	77.2

**Fig. 1:**
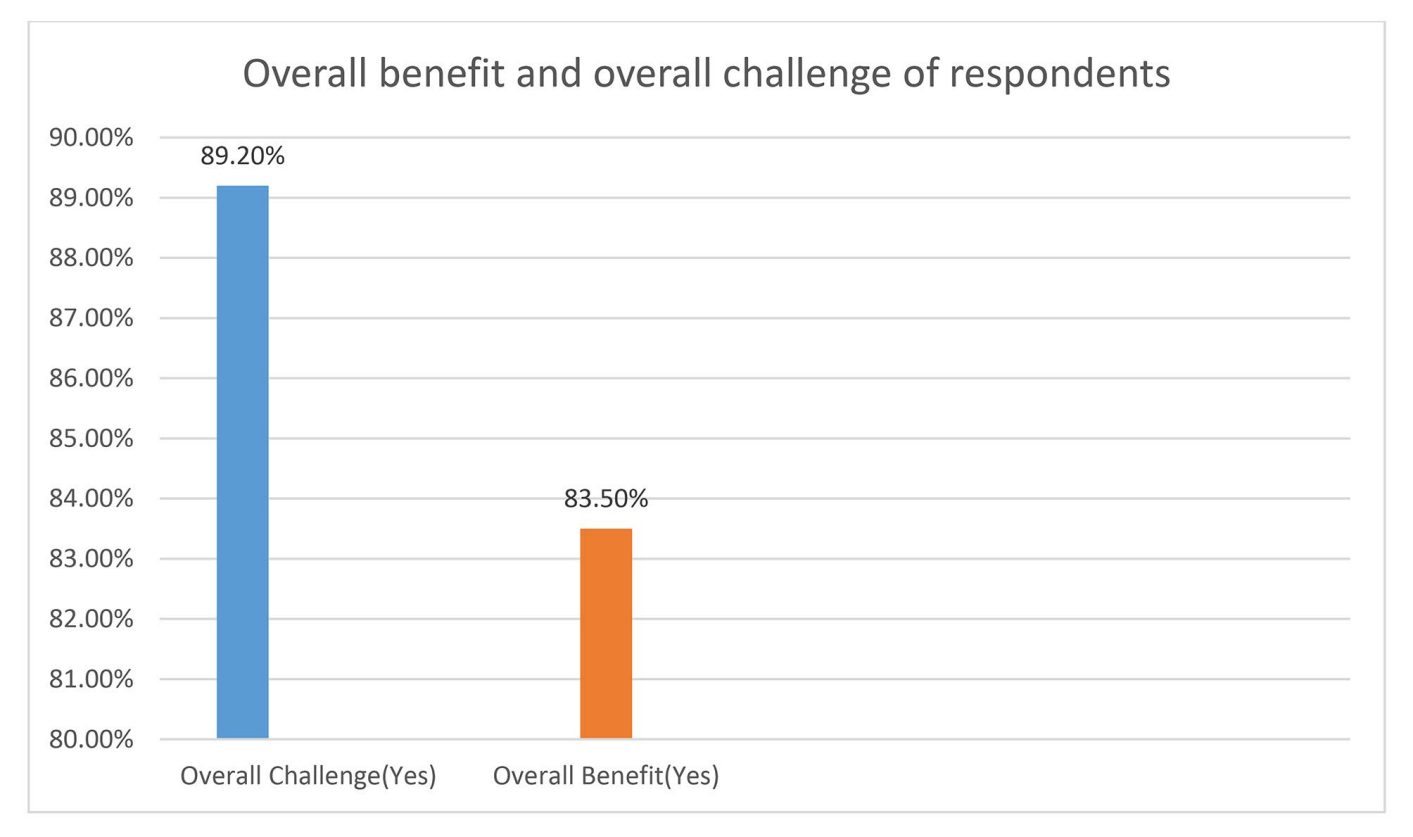
Overall benefit and overall challenge of NYSC among corps doctors

**Fig. 2:**
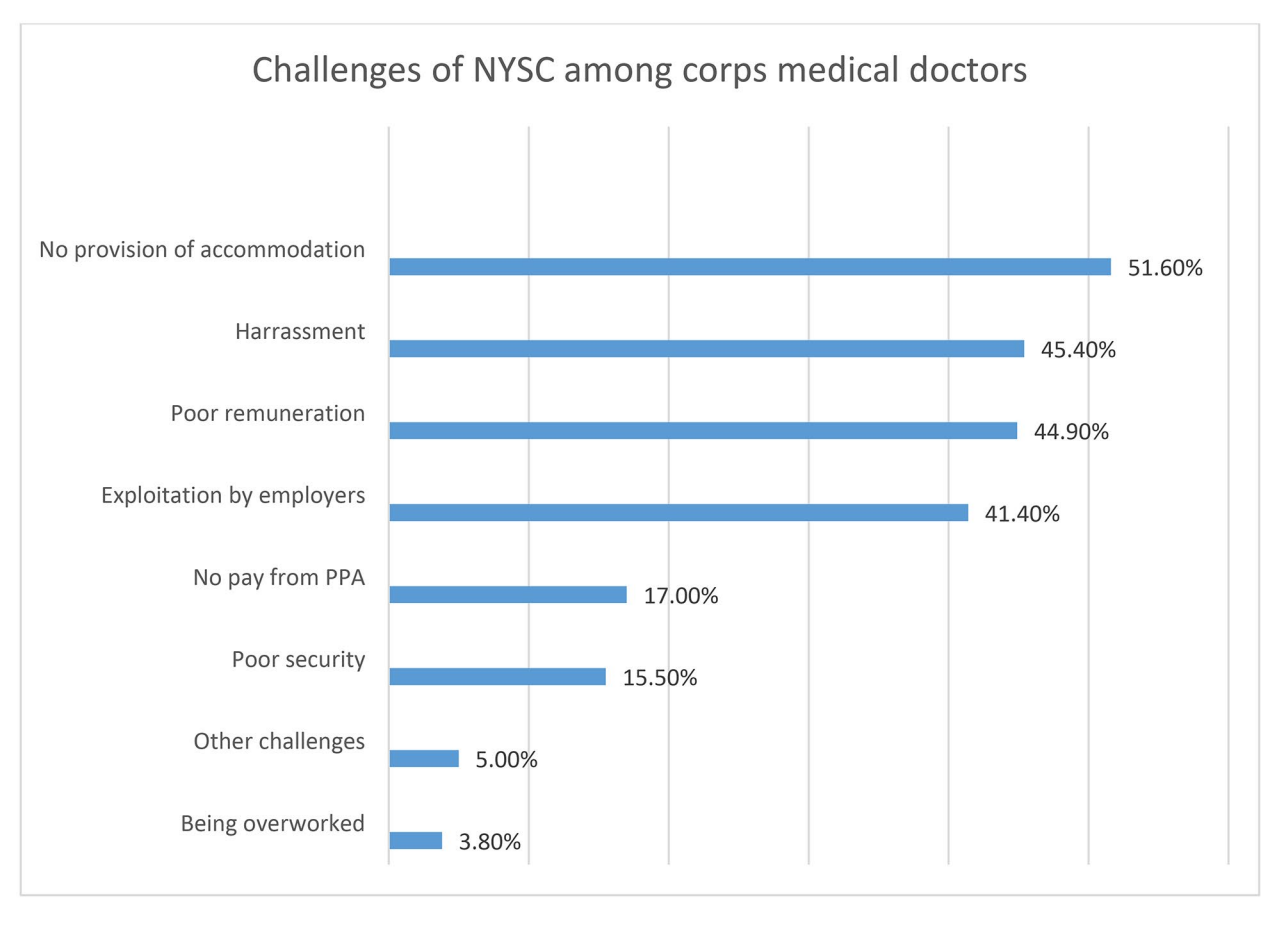
Challenges of NYSC among corps medical doctors

The proportion of corps doctors who reported one or more challenges was 89.2% (overall challenge) while the proportion of corps doctors who reported one or more benefits was 83.5% (overall benefit). Figure 1. The reported challenges were; no provision of accommodation (51.6%), harassment (45.4%), poor remuneration (44.9%), exploitation of corps doctors by employers (41.4%), poor security (15.5%) and being overworked (3.8%). Figure 2.

**Fig. 3:**
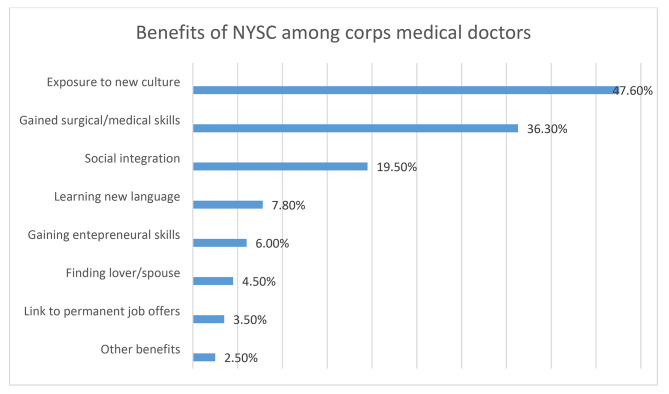
Benefits of NYSC among corps medical doctors

The reported benefits were; better exposure to new culture and new life (47.6%), gaining surgical/medical skills (36.3%), social integration (19.5%), learning a new language (7.8%), entrepreneurial skills (6.0%) and finding lover/spouse (4.5%). Figure 3.

Region of deployment had a statistically significant association with overall benefits (P- 0.013) as a slightly higher proportion of corps doctors serving in the north reported at least one benefit compared to their counterparts in the south. There was a statistically significant association between overall challenge and region of deployment (P- 0.031) and marital status (P- <0.001) as a higher proportion of corps doctors serving in the south and single corps doctors reported one or more challenges. Table 3.

**Table 3:** Association between socio-demographics and overall challenge and benefit

Variable	Overall benefit (n = 399)			Overall challenge (n = 399)		
	Yes N (%)	No N (%)	P value	Yes N (%)	No N (%)	P value
**Region of deployment**			0.013			0.031
North	206(87.3)	30(12.7)		204(86.4)	32(13.6)	
South	127(77.9)	36(22.1)		152(93.3)	11(6.7)	
**Medical school location**			0.391			0.846
Abroad	56(80)	14(20)		62(88.6)	8(11.4)	
Nigeria	277(84.2)	52(15.8)		294(89.4)	35(10.6)	
**Sex**			0.381			0.831
Male	171(85.1)	30(14.9)		180(89.6)	21(10.4)	
Female	162(81.8)	36(18.2)		176(88.9)	22(11.1)	
**Marital status**			0.266			< 0.001
Married	59(88.1)	8(11.9)		50(74.6)	17(25.4)	
Single	274(82.5)	58(17.5)		306(92.2)	26(7.8)	
**Tribe**			0.855			0.328
Hausa	56(80.0)	14(20.0)		60(85.7)	10(14.3)	
Igbo	89(84.8)	16(15.2)		98(93.3)	7(6.7)	
Yoruba	77(83.7)	15(16.3)		83(90.2)	9(9.8)	
Others	111(84.1)	21(15.9)		115(87.1)	17(12.9)	
**Religion**			0.535			0.341
Christian	225(84.3)	42(15.7)		241(90.3)	26(9.7)	
Islam	108(81.8)	24(18.2)		115(87.1)	17(12.9)	
**Age (years)**			0.142			0.272
23–29	292(82.5)	62(17.5)		318(89.8)	36(10.2)	
30 and above	41(91.1)	4(8.9)		38(84.4)	7(15.6)	
**Duration in service**			0.638			0.470
Less than 6 months	121(82.3)	26(17.7)		129(87.8)	18(12.2)	
6 months and above	212(84.1)	40(15.9)		227(90.1)	25(9.9)	
**PPA Type**			0.105			0.297
Government	270(82.1)	59(17.9)		296(90.0)	33(10.0)	
Non-Government	63(90.0)	7(10.0)		60(85.7)	10(14.3)	

More Christian corps doctors and single corps doctors reported bad treatment at their PPA when compared to their Islam and married counterparts respectively and religion and marital status had a statistically significant association with treatment at PPA with P values of 0.024 and 0.002 respectively. Table 4.

**Table 4:** Association between socio-demographics and Overall Treatment at PPA

Variable	Overall Treatment at PPA (n = 399)		
	Good N (%)	Bad N (%)	P value
**Region of deployment**			0.134
North	60(25.4)	176(74.6)	
South	31(19.0)	132(81.0)	
**Medical school location**			0.114
Abroad	21(30.0)	49(70.0)	
Nigeria	70(21.3)	259(78.7)	
**Sex**			0.163
Male	40(19.9)	161(80.1)	
Female	51(25.8)	147(74.2)	
**Marital status**			0.002
Married	25(37.3)	42(62.7)	
Single	66(19.9)	266(80.1)	
**Tribe**			0.710
Hausa	19(27.1)	51(72.9)	
Igbo	23(21.9)	82(78.1)	
Yoruba	18(19.6)	74(80.4)	
Others	31(23.5)	101(76.5)	
**Religion**			0.024
Christian	52(19.5)	215(80.5)	
Islam	39(29.5)	93(70.5)	
**Age (years)**			0.302
23–29	78(22.0)	276(78)	
30 and above	13(28.9)	32(71.1)	
**Duration in service**			0.390
Less than 6 months	37(25.2)	110(74.8)	
6 months and above	54(21.4)	198(78.6)	
**PPA Type**			0.538
Government	77(23.4)	252(76.6)	
Non-Government	14(20.0)	56(80)	

There were some recommendations from the respondents and almost half of the corps doctors (42.9%) opined that the NYSC scheme should be made optional. Table 5.

**Table 5:** Recommendation from respondents

Variable	Response	Frequency (N = 399)	Percentage (%)
What should be done to NYSC	Made optional	171	42.9
	Scrapped for doctors only	61	15.2
	Scrapped totally	94	23.6
	Revamped	73	18.3

## Discussion

A 15-year review of the compulsory community service scheme for South African medical doctors showed that two-thirds of respondents in their study expressed satisfaction with their accommodation and general welfare [23], as opposed to 89.2% of respondents in our study reporting one or more welfare-related problems such as exploitation, poor remuneration and lack of accommodation at their PPAs. These perceptual differences may be rooted in the fact that better welfare packages are made available for young South African doctors involved in the community service scheme, and imply that drastic improvement in welfare for corps medical doctors in Nigeria may go a long way in positively influencing their participation in the scheme.

More than half of our respondents (53.9%) were not deployed to any of their preferred states and about 80% of them reported dissatisfaction with their job at their PPAs. This is at variance with a similar study done in Ghana where more than 80% of the participants were posted to their preferred regions in Ghana and most of them enjoyed their service [22]. The difference in results might be related to the difference in posting styles in addition to the difference in study location. It can thus be inferred that being posted to a region of one’s preference might better improve the level of satisfaction of corps medical doctors during the service year.

NYSC has been seen by employers as a source of cheap labour whereby corps members are offered paltry stipends which are not commensurate with the rendered services [9]. Corps doctors are not left out of this unfair treatment. According to our study, most corps doctors (66.9%) receive less than 80,000 from their PPAs while 17% of corps doctors were not paid a dime at their PPAs. This is against the directives of the National Salaries, Income and Wages Commission of Nigeria on the allowances corps medical doctors are supposed to receive [24]. This gross reduction in earning potential of medical doctors during the period of national service is a significant challenge faced by the majority, and may prove to foster a negative perception of the scheme among corps doctors. This may eventually result in a significant fall in the participation of medical doctors in the scheme, with far-reaching consequences for the communities served by this group of professionals all over the country.

From our study, 15.5% of respondents cited poor security as part of the challenges of participating in the scheme. This is close to the findings of a study done among corps members in Abuja, Nigeria where 10% of participants reported that insecurity was a problem facing the scheme [25]. The relatively higher percentage from our study could be due to the difference in sample size and sample population as the above study was conducted among corps members in Abuja, the nation’s capital which is relatively safe.

This study also reported the experiences of corps doctors at their places of primary assignment as 77.2% of respondents reported overall bad treatment at their PPA. Problems faced at PPAs include no payment (17.0%), no accommodation (51.6%), exploitation (41.4%), insecurity (15.5%). Although our study was carried out among corps doctors from all states in Nigeria, a study done among corps members in Ibadan, Nigeria also reported similar problems: accommodation problems (79.7%), insecurity (79.7%) [4]. The study was conducted among 177 corps members from different fields in Ibadan, Nigeria so the differences in sample size, distribution and population could account for the observed differences. These statistics reflect the need for the leadership of the NYSC to be more proactive in the monitoring of the welfare of corps members posted to various PPAs.

About half of the respondents in a study done among corps members in Abuja, Nigeria reported finding a spouse from the host communities where they served as a benefit of NYSC. However, only 4.5% of our respondents reported finding a lover or spouse through the scheme as a benefit. This lower percentage reported in our study may be related to the difference in sample size as only 57 respondents were involved in the study [25].

Our study revealed that only 20.6% of corps doctors were happy with their job and this was similar to the finding of a study done among corps members in Bayelsa State, Nigeria where only 17% of respondents reported being happy with their job [26]. Although unlike our study, the study population comprised corps members in various fields, the similarity in results could imply a similar level of dissatisfaction with NYSC jobs between corps medical doctors and corps members in other fields. This low level of job satisfaction could be a consequence of the numerous highlighted challenges being faced by corps members, including those which are unique to serving medical doctors. More studies are needed to explore this phenomenon.

The proportion of corps medical doctors who reported challenges was higher among corps members serving in the south when compared to those serving in the north (93.3% compared to 86.4%) and this finding was noted to be statistically significant. Also, a higher proportion corps doctors serving in the northern regions were noted to report an overall good treatment at their PPAs when compared to their southern counterparts. This slight difference may be explained by the relative scarcity of medical doctors in the north, as the northern region of Nigeria has a low doctor-to-patient ratio (1 to 45,000) compared to the southern region (1 to 30,000) [27]. This fact may motivate employers and community members in northern Nigeria to treat the few doctors that they get access to with more reverence and hold them in higher regard.

From our study, 23.6% of respondents stated that the programme, NYSC should be scrapped completely, while 18.3% suggested that the programme be revamped. Almost half (42.9%) of our respondents suggested that the national service be made optional. These opinions suggest that the programme needs attention. Exhaustive and holistic assessment of the national service programme, with the implementation of befitting changes could grossly improve the perception of participants, including medical doctors.

### Limitations of the study

The limitations of the study include recall bias from the respondents. Also, poor internet connectivity is still a pervasive problem in Nigeria. Being an online survey, corps doctors serving in areas with poor internet connectivity may have been inadvertently excluded from the study as their responses may not have been transferred to our database.

## Conclusion and recommendation

This study showed that even though NYSC programme offers some benefits to corps medical doctors, there are also some challenges being faced by this group of professionals participating in the scheme, which may negatively influence their perception of the programme. There is a significant lack of data on the experiences of these doctors while participating in the scheme. It is recommended that the scheme be reviewed by relevant stakeholders and a favourable framework be developed for corps doctors, taking into consideration all of the challenges that we have highlighted. It is recommended that corps members, especially doctors be allowed to assess their PPAs just as the PPAs are expected to evaluate and conduct clearance procedures for corps members every month. The report of the corps members on their PPAs would be utilized by NYSC in the analysis of PPAs and subsequent posting of corps members. The NYSC should ensure the implementation of the instructions on the PPA posting letters by various PPAs. A work schedule template should be developed for corps doctors and adopted at various PPAs to avoid undue exploitation. The government should take active steps to improve the remuneration of corps medical doctors. This will improve welfare, and by extension, improve service delivery to the patients and the communities in which they are serving their fatherland.

## Electronic supplementary material

Below is the link to the electronic supplementary material.

Supplementary Material 1

## Data Availability

The datasets used and analyzed during the current study are included in this published article and its supplementary files.

## List of abbreviations

NYSC: National Youth Service Corps
PPA(s): Place(s) of Primary Assignment
